# Fast-Growth Polymer: Fullerene Bulk-Heterojunction Thin Films for Efficient Organic Photovoltaics

**DOI:** 10.3390/nano14060502

**Published:** 2024-03-11

**Authors:** Daewon Chung, Chandran Balamurugan, Byoungwook Park, Hyeonryul Lee, Ilhyeon Cho, Chaerin Yoon, Soyeon Park, Yong-Ryun Jo, Joonhyeon Jeon, Soonil Hong, Sooncheol Kwon

**Affiliations:** 1 Department of Advanced Battery Convergence Engineering, Dongguk University, Seoul 04620, Republic of Korea; jung1362@dgu.edu (D.C.); rjaeh0321@gmail.com (H.L.); ehrehdmldhkd@dgu.ac.kr (I.C.); kkkuu1615@dgu.ac.kr (C.Y.); parkso729@dgu.ac.kr (S.P.); memory@dongguk.edu (J.J.); 2 Department of Energy and Materials Engineering, Dongguk University, Seoul 04620, Republic of Korea; cbalamurugan2008@gmail.com; 3 Division of Advanced Materials, Korea Research Institute of Chemical Technology, Daejeon 34114, Republic of Korea; pbw0531@krict.re.kr; 4 Electron Microscopy Laboratory, Advanced Institute of Instrumental Analysis (GAIA), Gwangju Institute of Science and Technology (GIST), Gwangju 61005, Republic of Korea; yrjo@gm.gist.ac.kr

**Keywords:** organic solar cells, bulk heterojunction, homogeneous morphology, P3HT (Poly-3-hexylthiophene), PCBM ([6,6]-phenyl-C61-butyric acid methyl ester)

## Abstract

The bulk-heterojunction (BHJ) system that uses a π-conjugated polymer as an electron donor, and a fullerene derivative as an electron acceptor, is widely used in organic solar cells (OSCs) to facilitate efficient charge separation and extraction. However, the conventional BHJ system still suffers from unwanted phase segregation caused by the existence of significant differences in surface energy between the two BHJ components and the charge extraction layer during film formation. In the present work, we demonstrate a sophisticated control of fast film-growth kinetics that can be used to achieve a uniform distribution of donor and acceptor materials in the BHJ layer of OSCs without undesirable phase separation. Our approach involves depositing the BHJ solution onto a spinning substrate, thus inducing rapid evaporation of the solvent during BHJ film formation. The fast-growth process prevents the fullerene derivative from migrating toward the charge extraction layer, thereby enabling a homogeneous distribution of the fullerene derivative within the BHJ film. The OSCs based on the fast-growth BHJ thin film are found to exhibit substantial increases in J_SC_, fill factor, and a PCE up to 11.27 mA/cm^2^, 66%, and 4.68%, respectively; this last value represents a remarkable 17% increase in PCE compared to that of conventional OSCs.

## 1. Introduction

Organic solar cells (OSCs), which are created based on composites of an electron-donating conjugated polymer and an electron-accepting fullerene, are considered to hold promise as a low cost, printable, portable, and flexible energy source for use in the near future [1,2,3,4]. There have been extensive efforts made over the past few decades to improve the associated materials and the device science, which has led to encouraging progress, with power conversion efficiencies approaching ~19% [5,6,7]. Although the performance of OSCs has steadily improved, there is still a need for further improvements in efficiency for large-scale commercialization. This class of devices uses the bulk-heterojunction (BHJ) concept, which involves a self-assembly of nanoscale heterojunctions via spontaneous phase separation of the donor–polymer and the acceptor–fullerene [8,9,10]. As a result, charge-separating donor–acceptor interfaces are formed throughout the bulk of the composite material, thus resulting in a noble system with both efficient charge separation (by inhibiting early time recombination), and charge collection (by forming interpenetrating networks to the electrodes) [11,12]. 

To this end, recent research has introduced molecular doping, which is one of the strategies that can be used to optimize the bulk-heterojunction system. Although there is still a lack of understanding of the BHJ system to which the doping concept is applied, this strategy has been reported to lead to improved performance results [13,14,15]. Moreover, the optimal nanoscale morphology within the BHJ photoactive layer plays a critical role in facilitating efficient exciton dissociation and charge transport, which is primarily attributed to the short diffusion length of excitons (approximately 10 nm) in organic semiconductor materials [16,17]. The configuration of the BHJ photoactive layer primarily depends on factors such as the characteristics of the donors and the acceptors, the film processing techniques used, and the device structure. Significant improvements have been shown with the development of novel semiconducting polymers such as poly(thieno [3,4-b]thiophene-alt-benzodithiophene) derivative (PTB7-Th) and PM6, along with the non-fullerene acceptor Y6 [18,19,20]. Processes involving additive techniques, such as the use of a small amount of high boiling point solvent and organic molecules, has emerged as an effective approach for manipulating the nanoscale morphology of BHJ in state-of-the-art OSCs, and this approach has been shown to lead to enhanced PCEs [21,22].

For more efficient charge separation and collection, the rich region of donor polymer should be close to the interface of the HTL, while the rich region of fullerene derivative should be close to the ETL and/or cathode [23,24,25]. With the BHJ architecture, it is essential to implement rigorous control of the donor–acceptor morphology to achieve high-performance OSCs, and there has been a significant research focus on optimizing the donor–acceptor morphology. However, it has recently been reported that OSCs fabricated using conventional spin-coating methods do not exhibit an ideal donor–acceptor morphology of the photoactive layer. The BHJ layers have been reported to show an inhomogeneous distribution of the acceptor (fullerene) along the direction perpendicular to the substrates, with a higher density near the hole transport layer (HTL) [26]. This unbalanced donor–acceptor ratio induces acceptor-clusters near the HTL, and it reduces the device efficiencies by causing the recombination of the generated charge carriers and/or inhibiting efficient hole transport to the anode [27,28,29,30,31]. Therefore, to maximize the effect of the donor–acceptor BHJ system, it is important to develop a new film-processing technology that can produce a homogeneous donor–acceptor morphology in BHJ films.

Here, we have demonstrated a new approach to the homogenous donor–acceptor morphology in the active layer, using a noble film casting method involving fast-growth, and we obtained an improved power conversion efficiency (PCE) with an enhanced internal quantum efficiency of the device. The OSCs derived using the fast-growth method showed a higher value for J_SC_ of 11.27 mA/cm^2^, and a higher fill factor of 66% compared to the conventional one, which led to an overall increase in PCE to 4.68%. The results showed that the homogeneous donor–acceptor morphology has been successfully optimized to the BHJ system, which can therefore lead to the highly efficient performance of OSCs.

## 2. Materials and Methods

Device Fabrication: OSCs were fabricated on an indium tin oxide (ITO)-coated glass substrate with the following structure—ITO-coated glass substrate /Poly (3,4-ethylenedioxythiophene) (PEDOT:PSS)/Poly (3-hexylthiophene) (P3HT):Phenyl-C61-butyric acid methyl ester (PCBM)/Aluminum. Next, the ITO-coated glass substrate was cleaned with detergent; ultrasonicated in de-ionized water, acetone, and isopropyl alcohol for 10 min each; and subsequently dried overnight in an oven. PEDOT:PSS (Clevious PH) was then deposited on the ITO-coated glass substrate to form a 20 nm-thick film. At this point, the substrate was dried at 140 °C for 10 min in air. Then, the P3HT:PCBM BHJ layer was spun on the PEDOT:PSS substrate at various spin speeds in a glove box filled with N2. The solution of the BHJ layer was a mixture of P3HT:PCBM (1:0.7) in chloroform solvent with a concentration of 17 mg/mL (1 wt%). The film was dried for 10 min at 80 °C in the glove box. By changing the spin-coating rpm from 2000 rpm to 8000 rpm, the thickness was controlled from 150 nm to 80 nm. Figure S1 depicts the procedure of both the conventional and fast-growth coating methods of the P3HT:PCBM photoactive layer. Then, an aluminum (Al, 100 nm) electrode was deposited through thermal evaporation in a vacuum of about 5 × 10^−6^ torr. The final devices were annealed for 10 min at 150 °C in a glove box filled with N2. To prepare the PM6:Y6 photoactive layer-based OSCs, the same procedure was conducted from the ITO-coated glass substrate cleaning to the spin-coating of the PEDOT:PSS. The PM6:Y6 BHJ layer was spin-coated on the PEDOT:PSS substrate in a glove box filled with N2. The PM6 and Y6 mixture was dissolved in chloroform solvent at a ratio of 1:1.6 and a concentration of 13 mg/mL. After PM6:Y6 coating, the substrate was dried at room temperature for 5 min. The ZnO nanoparticle (2.5% in mixed alcohol; Nano Clean Tech) solution was then diluted in isopropanol to 1%. The ZnO nanoparticle solution was spin-coated at 3000 rpm for 30s on top of the PM6:Y6 photoactive layer. Then, a silver (Ag, 100 nm) electrode was deposited.

Characterization: The current–voltage (J–V) characteristics of the devices were measured using a Keithley 236 Source Measure Unit. To measure the performance of the OSCs, an Air Mass 1.5 Global (AM 1.5 G) solar simulator was used with an irradiation intensity of 100 mW/cm^2^, which produces a homogeneous donor–acceptor morphology in the BHJ films. UV-vis absorption spectra were obtained using a UV-vis-NIR spectrometer (Lambda750UV-vis-NIR spectrometer, Perkin-Elmer Korea Ltd. Co., Seoul, Republic of Korea). External quantum efficiency (EQE) was obtained using a Solar Cell Spectral Response/QE/IPCE measurement system (PV Measurements Inc., Washington, USA) with a chopping frequency of 100 Hz. Three-dimensional atomic force microscopy (AFM) images were obtained using a Park System with an XE-100 microscope.

TEM microscopy: Slices were prepared by casting a P3HT:PCBM blend thin film on ITO-covered glass. The films were then baked at 150 °C for 10 min. Pt was deposited on the surface to protect it from contamination by other organic materials. After rough and fine milling on the BHJ film with a focused ion beam (Vacc. 30 keV), the slice was picked up and transferred to a copper mesh TEM grid with a carbon coating. Light field imaging was performed in a FEI (Tecnai F30 Super-twin) TEM using proper defocus for additional phase contrast from the relatively amorphous polymer material.

## 3. Results and Discussions

To this point, there have been many studies examining the morphology of the donor–acceptor network composites. Prior researchers have found that the difference in the surface energy, $\gamma_{s}$, of each material determines the free energy of the system, which tends toward minimization, and this causes the morphology in the BHJ film to significantly depend on the difference in the surface energy between the donor–acceptor composites and the HTL [32,33,34]; thus, a heterogeneous morphology of components can be formed at the buried interface if the donor–acceptor composites in the BHJ film have surface energies that are substantially different from that of the HTL. This means that if the surface energies of the donor–acceptor composites substantially deviate from that of the HTL, it can lead to non-uniform distribution and segregation of materials at the interface. Such heterogeneity can have profound implications for the performance of OSCs, as it directly impacts charge transport and recombination dynamics. Therefore, understanding and controlling surface energy disparities is crucial for engineering optimized morphologies and ultimately enhancing device performance.

In terms of morphology, we consider a well-known BHJ solar cell, ITO glass substrate /Poly(3,4-ethylenedioxythiophene) (PEDOT:PSS)/Poly-3-hexylthiophene (P3HT): Phenyl-C61-butyric acid methyl ester (PCBM) /Aluminum, while considering the different surface energies ($\gamma_{s}$) of PEDOT:PSS ($\gamma_{s}$ = 47.5 mN/m), PCBM ($\gamma_{s}$ = 38.2 mN/m), and P3HT ($\gamma_{s}$ = 26.9 mN/m) [35,36,37]. As shown in Figure S2, the P3HT and PCBM exhibited a high contact angle of 101.3° and a low contact angle of 83.4°, respectively, indicating that PCBM as an acceptor has a higher surface energy than that of the donor, P3HT, and that the $\gamma_{s}$ of PCBM is consequently closer to that of PEDOT: PSS. Therefore, during spin-coating using the P3HT: PCBM blend, a PCBM-rich region can be expected to occur at the buried interface between PEDOT: PSS and the BHJ layer. Moreover, if a PCBM-rich region is formed on the surface of PEDOT:PSS once, the region cannot be mixed again or removed, even with a high spin speed. Eventually, the accumulated PCBM at the interface leads to a heterogeneous morphology (which is often meta-stable) in the BHJ layer, which causes a problem for the hole carrier transfer from the BHJ layer to PEDOT:PSS.

**Figure 1:**
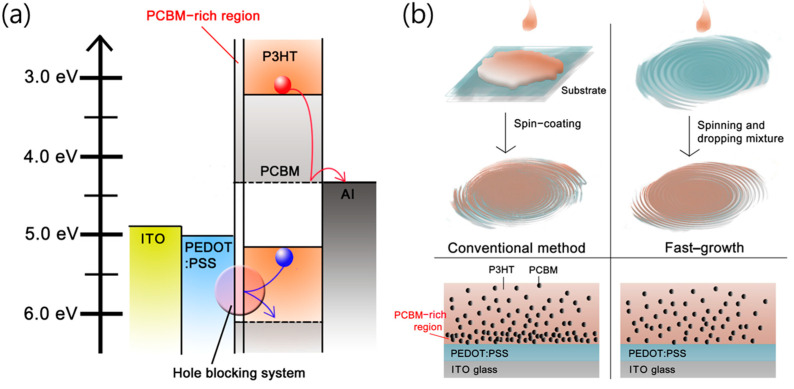
(**a**) Energy band diagram of conventional cell. (**b**) Schematic procedure of two different methods, conventional and fast-growth.

Figure 1a depicts an energy-level diagram of a conventional BHJ solar cell that has the PCBM-rich region. In this case, the PCBM-rich region at the interface between the PEDOT:PSS and BHJ layer acts as a hole-blocking system, thus inhibiting the hole carrier from transporting to PEDOT:PSS. The blocked charges increase the probability of recombination in the BHJ layer, which directly reduces the photovoltaic efficiency. Although Z. Xu et al. suggested that an inverted structure was an ideal permutation due to the PCBM-rich region, which is close to the cathode, ITO, it is still difficult to achieve high-performance OSCs, since there is a mismatching problem of the work function of the electrode, and a processing problem because of surface properties between layers [38,39]. To overcome the problem of the heterogeneous morphology in the BHJ layer, we have introduced a homogeneous morphology in the BHJ layer using the fast-growth coating method, which is quite different in the time scale of the film formation. In this method, three critical advantages can be expected from the fast-growth: (1) a homogeneous donor–acceptor network morphology in the BHJ layer; (2) control of overgrowing grain size; and (3) a smooth surface morphology of the BHJ layer. We have found that all of these advantages contribute to enhancing the performance of OSCs.

Figure 1b summarizes the differences between the fast-growth and the conventional coating process. For the conventional method, the mixed solution is deposited on the PEDOT:PSS layer, after which the PCBM-rich region is formed within only a few seconds because the components in BHJ film can move freely to the surface of PEDOT:PSS before a solvent evaporates. On the other hand, in the fast-growth case, when the substrate is spinning and then the mixed solution is dropped on it, a homogeneous film is achieved because of the rapid film formation of the film (~10^−3^ s). Moreover, because of the rapid film formation, we expect that PCBM does not have enough time to nucleate on the surface of the PEDOT:PSS. To directly obtain information about the network morphology of the donor–acceptor composites, we have measured cross-section images of the device using field-emission transmission electron microscopy (FE-TEM).

**Figure 2:**
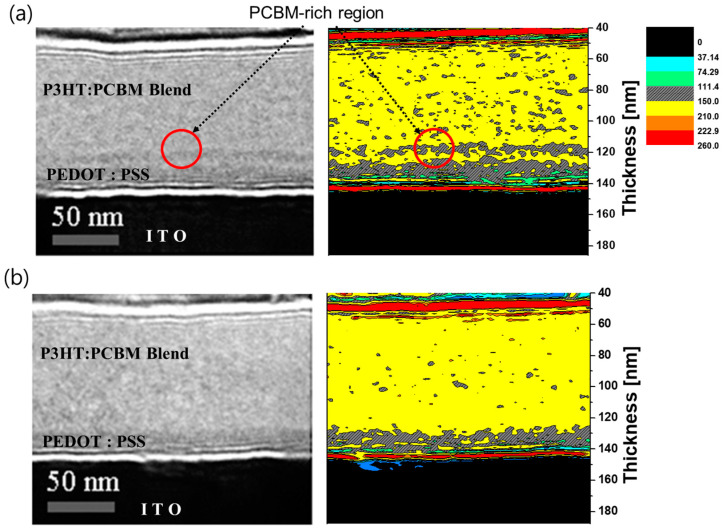
Defocused TEM (∆Z = −25 um) cross-sectional images and image profiling of the two different cells: (**a**) conventional and (**b**) fast-growth.

Figure 2 shows the defocused TEM cross-sectional images of the devices that were annealed at 150 °C for 10 min, which were fabricated using either the conventional method or the fast-growth method. Although the samples had the same device structure and thickness, we directly observed an additional electron-rich region (red circle) on the buried interface between PEDOT:PSS and the BHJ in the conventional cell due to the phase contrast of the different composites, with PCBM showing up as the darker region as it moved into the electron-rich region. A similar phenomenon was also detected in the conventional cell with a thick photoactive layer, as shown in Figure S2. On the other hand, in the fast-growth cell, there was no electron rich part at the surface of PEDOT:PSS. We thus confirmed that the PCBM-rich region is formed on the surface of PEDOT:PSS and that a homogeneous morphology can be successfully achieved using the fast-growth method. Further, through image profiling, which converts the intensity of darkness to various colors, we have noticed the control of not only a PCBM-rich region but also an overgrowing PCBM grain size.

**Figure 3:**
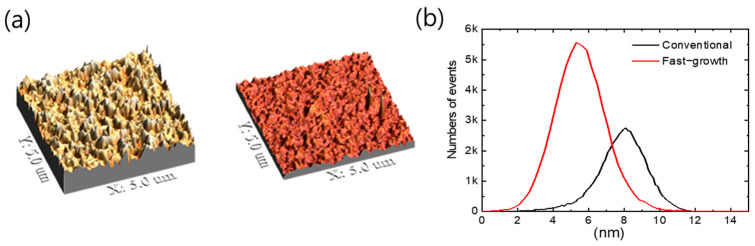
(**a**) 3-D AFM image of the conventional cell and the fast-growth cell, which are annealed at 150 °C for 10 min. (**b**) Curves showing the number of peaks in the surface of each device with height.

In addition to the two advantages of a homogeneous morphology and the control of an overgrowing PCBM grain size, we also note that the fast-growth cell has a smooth surface morphology that improves the adhesion at the interface between the BHJ layer and the aluminum electrode. To compare the surface morphology of each device, AFM measurement of each surface was conducted. Figure 3 shows surface images of the devices and the surface information with height. The 3-D AFM data showed a uniform and smooth surface morphology (~5 nm) in the fast-growth cell, while there was a relatively rough morphology (~8 nm) in the conventional cell. We expect that this difference in the surface morphology could be attributed to the rapid film formation of the BHJ layer. Moreover, the two-dimensional (2-D) AFM topography analysis corroborated the trends observed in the 3-D images, as depicted in Figure S4. These findings collectively suggest that the fast-growth cell not only achieves superior morphological control but also facilitates the formation of a smoother surface interface, thereby improving adhesion between the BHJ layer and the aluminum electrode. Such advancements in surface morphology are crucial for optimizing the performance of OSCs.

**Figure 4:**
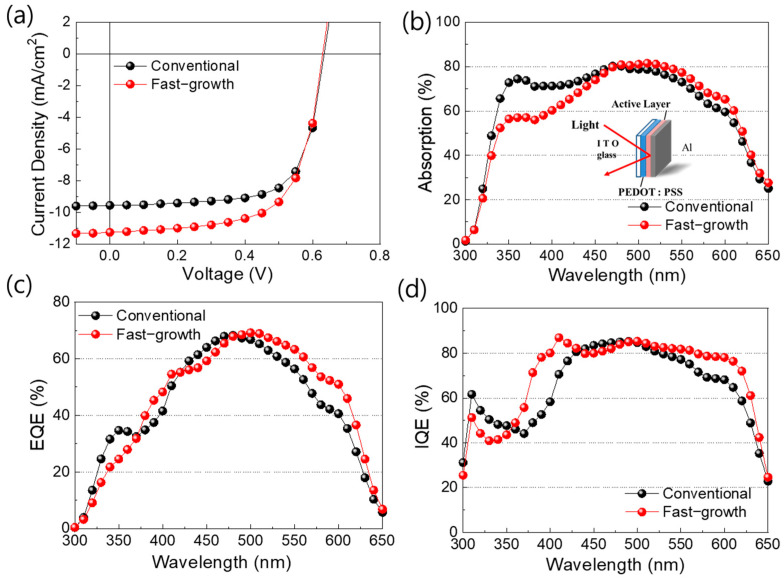
Comparison of various properties of the fast-growth and conventional cell. (**a**) J–V characteristics, (**b**) optical properties via reflectance geometry, (**c**) IPCE, and (**d**) IQE of the conventional and fast-growth cells.

One can expect the device prepared with the PCBM to be abundantly present around PEDOT:PSS, thus increasing the charge recombination, and thereby ensuring a decrease in the performance. For the realization of the phenomenon that the PCBM is heavily clustered on the surface of PEDOT:PSS, the C60 was thermally evaporated on top of the PEDOT:PSS layer with various thicknesses. Figure S5 and Table S1 show that a thicker C60 layer decreases the device performance due to the low shunt resistance and high series resistance. To compare performance among the different methods, we have measured the current-voltage (J–V) characteristics for the devices under AM 1.5 illumination from a solar simulator with an intensity of 100 mA/cm^2^. We then prepared the two devices with both a thin and a thick photoactive layer, as described previously, and optimized them to achieve the best performance (Figure S6 and Table S2). As shown in Figure 4a and Table 1, the J–V curves of the two devices reveal that the fast-growth cell is significantly different from the conventional cell in terms of short-circuit currents and fill factor, despite the fact that the two devices had almost the same thickness (150 nm) and V_oc_ (0.63 V). The fast-growth cell has a relatively higher fill factor, 66%, than that of the conventional one, 60%, and the difference in their short-circuit current amounts is also close to 1 mA/cm^2^. The two significantly higher values for J_SC_ (11.3 mA/cm^2)^, and fill factor (66%) in the fast-growth cell clearly led to the overall increase in PCE to 4.68%.

However, more clear evidence is required to prove how the homogeneous morphology affects the photovoltaic performance. Assuming a homogeneous morphology in the BHJ film and thus an easy pathway for the charge transfer, it is crucial to improve the internal quantum efficiency (IQE) of both the photoinduced electron and the photoinduced hole transfer. Enhancing the IQE of both photoinduced electron and hole-transfer processes involves optimizing various aspects of the device structure and materials. For instance, selecting donor and acceptor materials with complementary energy levels and suitable morphology can facilitate efficient exciton dissociation and charge carrier generation. Additionally, controlling the interface between the active layer and charge transport layers can minimize charge recombination and facilitate charge extraction. That is, the quantum efficiency of the fast-growth cell should not be dependent on the PCBM-rich regions as a hole-blocking system. To clarify the homogeneous morphology in the BHJ film, we have measured the optical absorption and EQE to calculate the internal quantum efficiency (IQE) [40,41,42]. The value of IQE directly reflects how efficiently the charge carrier travels to both electrodes as a function of wavelength, regardless of incident light. Therefore, the value of IQE serves as clear evidence for the homogeneous pathway in BHJ film, and it can explain the higher performance of the fast-growth cell [43,44].

Figure 4b shows the percentage of absorption spectra. To measure the exact amplitude of absorption in the BHJ layer, the absorption spectra were obtained by reflectance geometry to include the interference effect of the incident light with the metallic aluminum electrode. This reflected absorption is different from the general absorption related to optical density, shown in Figure S7. The fast-growth method shows lower absorption than the conventional method at wavelengths ranging from 325 nm to 450 nm. In the case of PCBM, the absorption spectrum exhibits pronounced absorption ranging from 300 to 500 nm, while for P3HT, absorption gradually intensifies starting around 350 nm and reaches a peak at 525 nm (Figure S8). This can be speculated to mean that PCBM tends to accumulate near PEDOT:PSS, thus leading to aggregation, and thereby highlighting the substantial influence of PCBM absorption within the 325 to 450 nm range in the conventional coating method. Conversely, the absorption peak appears comparatively diminished, which can be attributed to the reduced effect of the PCBM in the fast-growth coating method.

From the EQE data shown in Figure 4c, it could be observed that the value of the EQE of the device fabricated using the fast-growth method is much higher than that of the conventional device in the wavelength range from 500 to 650 nm. Using the absorption spectra and EQE data, we have calculated the IQE for each device, and we present these in Figure 4d. Aside from the noise in the wavelength range from around 300 to 350 nm, which is attributed to the UV cutting effect of glass substrate, the device fabricated with the fast-growth method has a constant value of IQE, 80%, in the entire wavelength range. Specifically, considering the maximum absorptions of PCBM (around 370 nm) and P3HT (around 520 nm), the internal quantum efficiency of both hole transfer by PCBM and electron transfer by P3HT in the fast-growth cell is dramatically increased in the wavelength range from 350 to 600 nm. The conventional cell also has a maximum IQE value of 80%, but only in a narrow wavelength range from 400 to 500 nm. These findings indicate that the heterogeneous morphology of the conventional cell clearly limits the IQE of both electron and hole transfer, while the homogeneous morphology of the fast-growth cell favors the transfer of the charge carriers generated at any wavelength. It is therefore expected that the improvements in the photocurrents and fill factor of the fast-growth cell are achieved by the highly efficient charge carrier collection over the broad wavelength range due to the homogeneous morphology. These results again indicate that the homogeneous morphology is a critical factor in achieving high performance.

**Figure 5:**
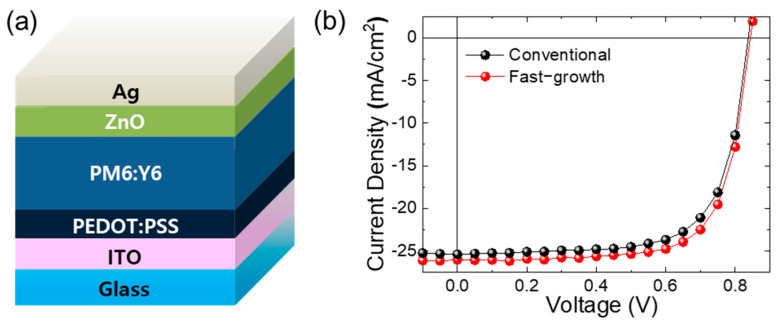
(**a**) Device structure of OSC using PM6:Y6 photoactive material and (**b**) J–V characteristics of the OSCs.

Further, OSCs based on the non-fullerene acceptor of PM6:Y6 have been tested to assess the generalizability of the fast-growth coating method. As shown in Figure S9, a vertical distribution or aggregation of Y6 toward the PEDOT:PSS surface is not found to be much like the P3HT:PCBM-based OSC in the defocused TEM analysis, and there were very slight increases in J_SC_, FF, and PCE (Figure 5 and Table 2). Because Y6 has a smaller bulky characteristic than PCBM, aggregation and the vertical distribution phenomenon are not severe even in the conventional coating method. Therefore, the increase in PCE is not distinct compared to that in the OSCs based on the P3HT:PCBM photoactive layer. Nevertheless, highly efficient OSCs were demonstrated by achieving values for J_SC_ of 26.0 mA/cm^2^ and for fill factor of 72% in the fast-growth cell, leading to an overall increase in PCE to 25.7%. While a more detailed processing method is necessary for the fabrication of highly efficient solar cells, these findings assert that it is crucial to achieve a morphology that is conducive to favorable energy levels.

## 4. Conclusions

In conclusion, we have demonstrated that the PCBM-rich region obviously limits the performance of OSCs in a conventional device, and that this problem can be solved by the homogeneous morphology achieved by the fast-growth method. Even though the procedure used to fabricate the fast-growth cell is almost identical to that used for the conventional one, the most important concept of the fast-growth method is its rapid film formation, which introduces a homogeneous morphology and thereby removes the PCBM-rich region. By introducing a homogeneous morphology in the BHJ layer, we obtain a much higher fill factor and increased short-circuit currents because of the easier transfer pathway for the charge carriers without the hole-blocking system. However, it should be noted that we do not believe that this study is limited to the typical OSC materials of P3HT and PCBM, as the potential for the fast-growth method was demonstrated to some extent in non-fullerene based solar cells made using PM6:Y6. That is, other materials that have different surface energies from each other should also be considered in view of the donor–acceptor morphology formation within the BHJ film. Although a more detailed processing method is needed to produce highly efficient OSCs, introducing a homogeneous morphology using the fast-growth method can be expected to lead to better performance of OSCs.

## Figures and Tables

**Table 1:** Photovoltaic performance parameters of OSCs using P3HT:PCBM photoactive material.

	J_SC_ (mA/cm^2^)	V_OC_ (V)	FF	PCE (%)
Conventional	10.3	0.63	0.60	3.88
Fast-growth	11.3	0.63	0.66	4.68

**Table 2:** Photovoltaic performance parameters of the OSCs using PM6:Y6 photoactive material.

	J_SC_ (mA/cm^2^)	V_OC_ (V)	FF	PCE (%)
Conventional	25.4	0.84	0.70	14.8
Fast growth	26.0	0.84	0.72	15.7

## Data Availability

Data are included in the article/supplementary materials/referenced in article.

## Supplementary Materials

The following supporting information can be downloaded at: https://www.mdpi.com/article/10.3390/nano14060502/s1, Figure S1: Schematic images of (a) conventional spin coating process and (b) fast-growth coating process; Figure S2: Contact angles of (a) P3HT and (b) PCBM films; Figure S3: (a) Cross-sectional TEM images of conventional cells with different thicknesses and fast-growth cell; Figure S4: AMF topography of P3HT:PCBM films with (a) conventional and (b) fast-growth coating method; Figure S5: (a) Device structure of fast-growth coating method on thermal evaporated C60; (b) J-V characteristic with various thickness of C60; Figure S6: (a) J-V characteristic of conventional and fast-growth OSCs with thin photoactive layer; Figure S7: Normalized optical density of OSCs coated using conventional coating method and fast-growth coating method; Figure S8: Normalized absorption spectra of P3HT and PCBM films; Figure S9: TEM and defocused Scanning TEM cross-sectional images of the PM6:Y6 OSCs with (a) conventional and (b) fast-growth coating method; Table S1: Shunt and series resistances of conventional cells and fast-growth cells with different thicknesses of C60; Table S2: Photovoltaic performance parameters of OSCs with thin photoactive layer.
