# Supplementary material for: Fast-Growth Polymer: Fullerene Bulk-Heterojunction Thin Films for Efficient Organic Photovoltaics
Source: Nanomaterials (Basel). 2024 Mar 11;14(6):502. doi: 10.3390/nano14060502 (PMC10975601; doi:10.3390/nano14060502)
Supplement: Supplementary file 1 [file nanomaterials-14-00502-s001.zip › nanomaterials-2884715-supplementary.pdf]

Supporting Materials

Fast-growth polymer:fullerene bulk-heterojunction thin films for efficient organic photovoltaics

Daewon Chung [1], Chandran Balamurugan [2], Byoungwook Park [3], Hyeonryul Lee [1], Ilhyeon Cho [1], Chaerin Yoon [1], Soyeon Park [1], Yong-Ryun Jo [4], Joonhyeon Jeon [1], Soonil Hong [3,*] and Sooncheol Kwon [2,*]

[1] Department of Advanced Battery Convergence Engineering, Dongguk University, Seoul 04620, Republic of Korea; jung1362@dgu.edu (D.C.); rjaeh0321@gmail.com (H.L.); ehrehdmldhkd@dgu.ac.kr (I.C.); kkkuu1615@dgu.ac.kr (C.Y.); parkso729@dgu.ac.kr (S.P.); memory@dongguk.edu (J.J.)
[2] Department of Energy and Materials Engineering, Dongguk University, Seoul 04620, Republic of Korea; cbalamurugan2008@gmail.com
[3] Division of Advanced Materials, Korea Research Institute of Chemical Technology, Daejeon 34114, Republic of Korea; pbw0531@krict.re.kr
[4] Electron Microscopy Laboratory, Advanced Institute of Instrumental Analysis (GAIA), Gwangju Institute of Science and Technology (GIST), Gwangju 61005, Republic of Korea; yrjo@gm.gist.ac.kr (Y.-R.J.)
* Correspondence: sihong@krict.re.kr (S.H.); kwansc12@dongguk.edu (S.K.)

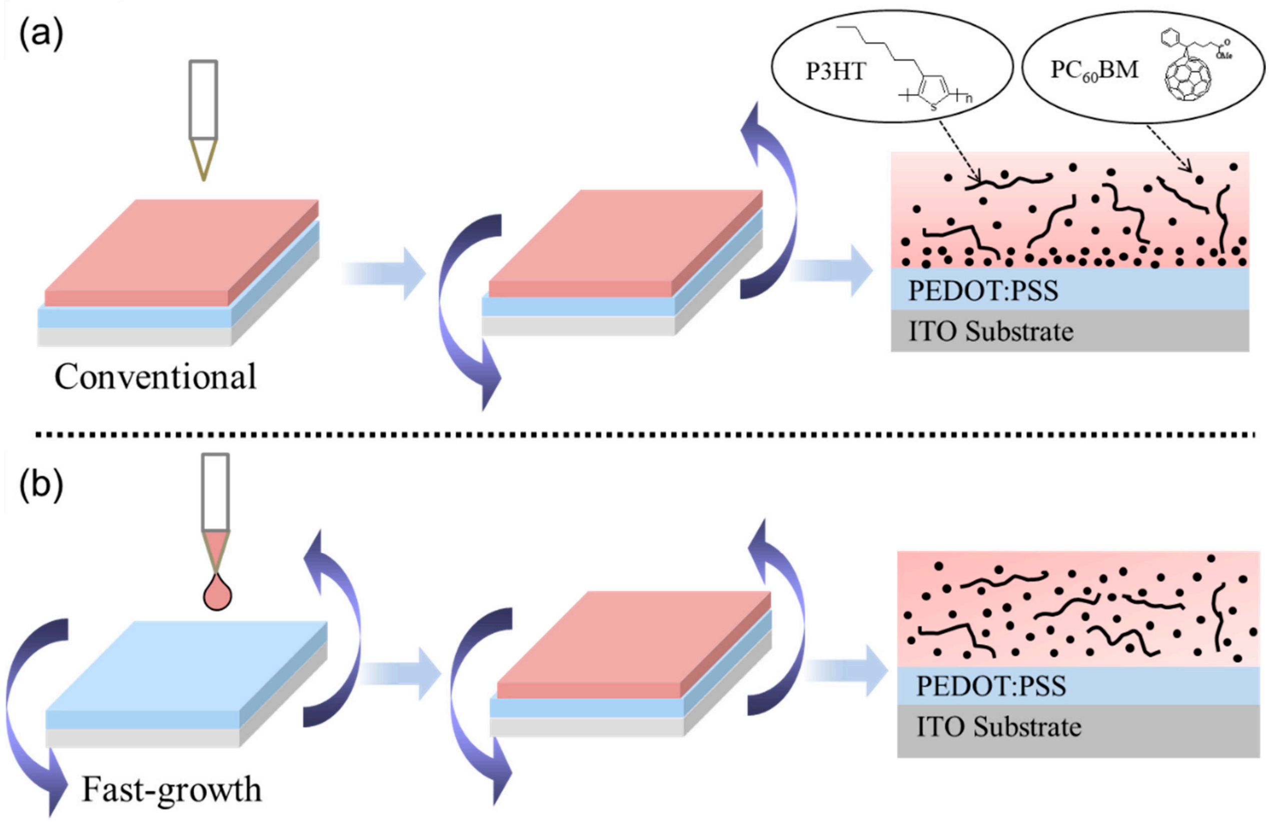

Figure S1. Schematic images of (a) conventional spin coating process and (b) fast-growth coating process.

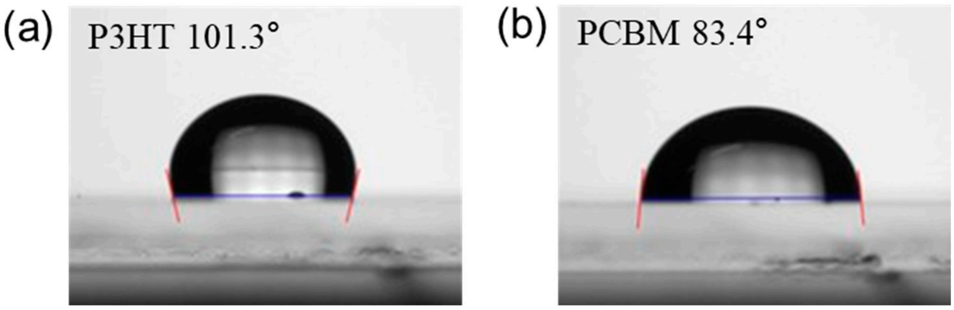

Figure S2. Contact angles of (a) P3HT and (b) PCBM films.

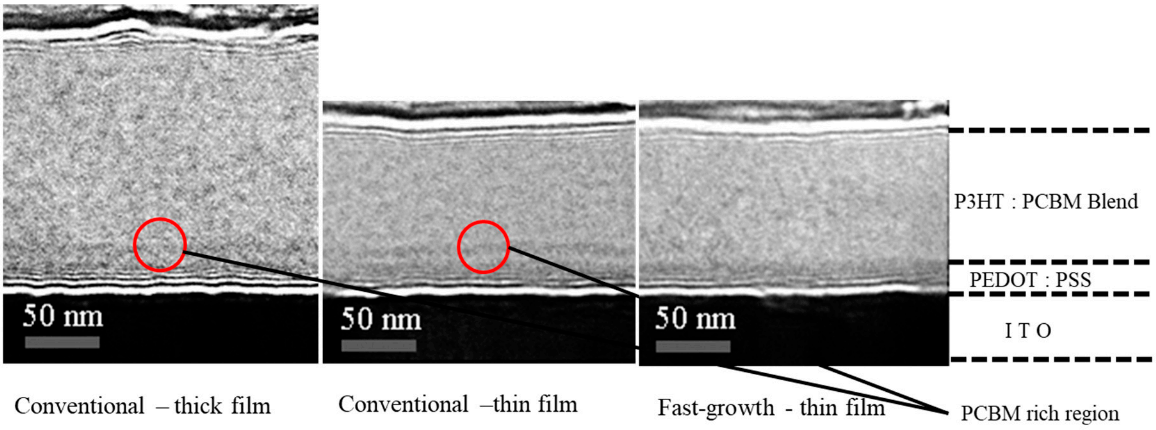

Figure S3. (a) Cross-sectional TEM images of conventional cells with different thicknesses and fast-growth cell.

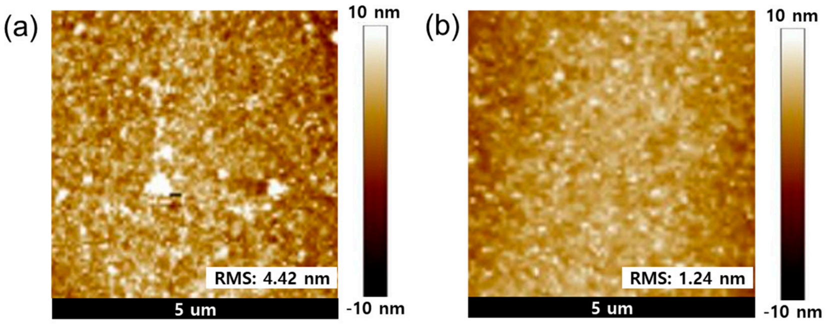

Figure S4. AMF topography of P3HT:PCBM films with (a) conventional and (b) fast-growth coating method.

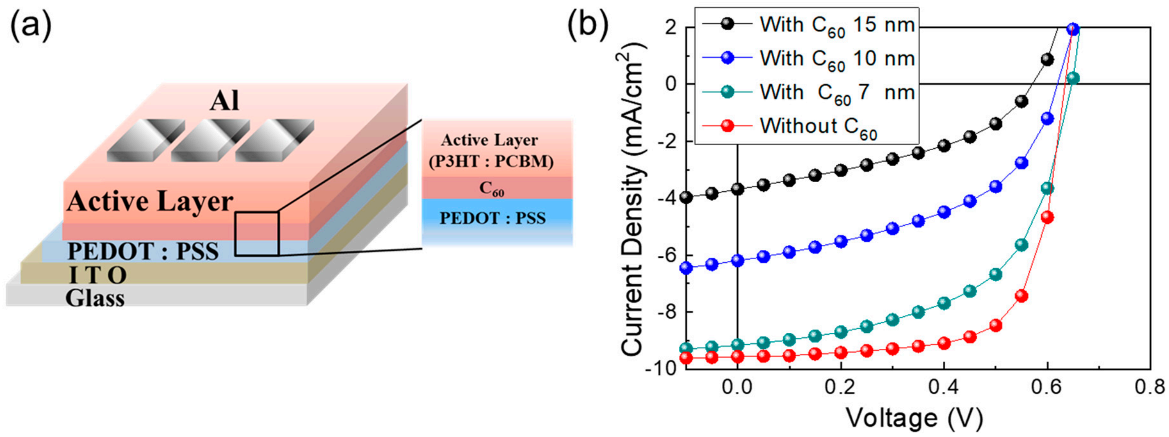

Figure S5. (a) Device structure of fast-growth coating method on thermal evaporated C_{60}; (b) J-V characteristic with various thickness of C_{60}.

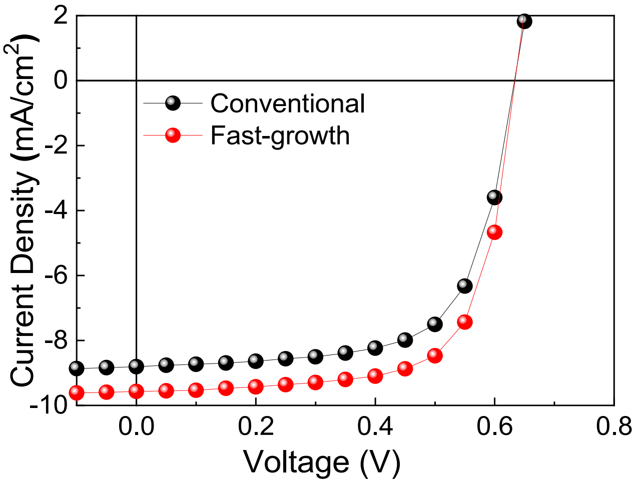

Figure S6. (a) J-V characteristic of conventional and fast-growth OSCs with thin photoactive layer.

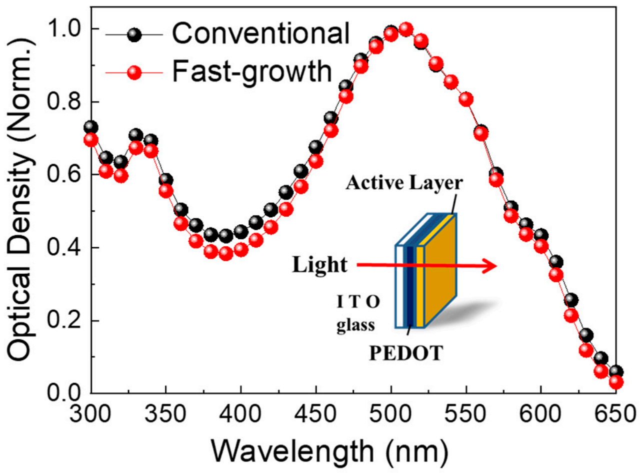

Figure S7. Normalized optical density of OSCs coated using conventional coating method and fast-growth coating method.

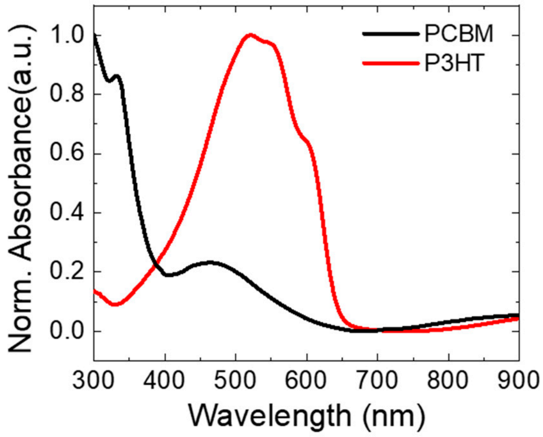

Figure S8. Normalized absorption spectra of P3HT and PCBM films.

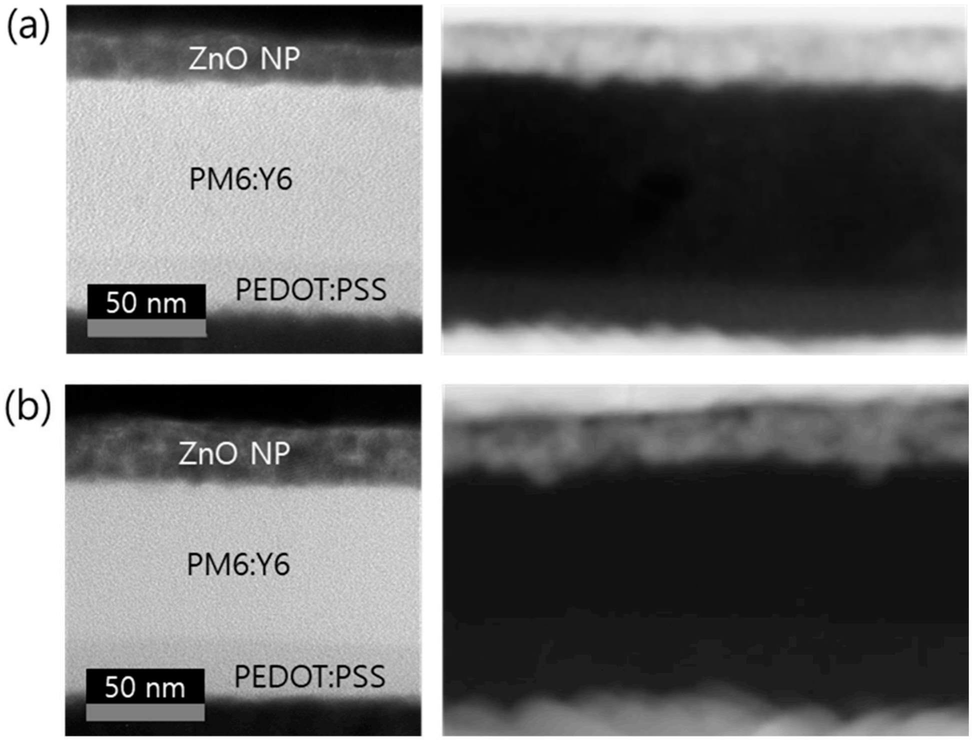

Figure S9. TEM and defocused Scanning TEM cross-sectional images of the PM6:Y6 OSCs with (a) conventional and (b) fast-growth coating method.

Table S1. Shunt and series resistances of conventional cells and fast-growth cells with different thicknesses of C_{60}.

	Shunt Resistance (Ω· cm^2)	Series Resistance (Ω· cm^2)
Fast-growth with C_{60} 15 nm	3.31	3.41 x 10^{-2}
Fast-growth with C_{60} 10 nm	3.69	1.60 x 10^{-2}
Fast-growth with C_{60} 7 nm	6.24	1.29 x 10^{-2}
Fast-growth	24.61	7.30 x 10^{-3}
Conventional	13.95	9.22 x 10^{-3}

Table S2. Photovoltaic performance parameters of OSCs with thin photoactive layer.

	J_{sc} (mA /cm^2)	V_{oc} (V)	FF	PCE (%)
Conventional	8.62	0.63	0.67	3.69
Fast-growth	9.57	0.63	0.70	4.24
